# The Use of Cognitive Cues for Anticipatory Strategies in a Dynamic Postural Control Task - Validation of a Novel Approach to Dual-Task Testing

**DOI:** 10.1371/journal.pone.0157421

**Published:** 2016-08-03

**Authors:** Uffe Laessoe, Bo Grarup, Jette Bangshaab

**Affiliations:** 1 Physiotherapy department, University College North Denmark, Denmark; 2 Research and Development department, University College North Denmark, Denmark; Ludwig-Maximilians-Universitat Munchen, GERMANY

## Abstract

**Introduction:**

Dual-task testing is relevant in the assessment of postural control. A combination of a primary (motor) and a secondary (distracting cognitive) tasks is most often used. It remains a challenge however, to standardize and monitor the cognitive task. In this study a new dual-task testing approach with a facilitating, rather than distracting, cognitive component was evaluated.

**Methods:**

Thirty-one community-dwelling elderly and fifteen young people were tested with respect to their ability to use anticipatory postural control strategies. The motor task consisted of twenty-five repetitive tasks in which the participants needed to exceed their limit of stability in order to touch one out of eight lights. The participants performed three tests. In two of the tests the color cues of the lights allowed the participants to utilize cognitive strategies to plan their next movement and improve their performance time.

**Results:**

The young performed the baseline motor task test in an average of 29 seconds, while the average time for the elderly was 44 seconds. When comparing the performance time with a leading cue to the time with no cue, the young group improved their performance time significantly better than the elderly did: young: 17% (5), elderly: 5% (8); p<0.001. Similar differences were seen with a more complicated leading cue: young: 12% (5), elderly: 4% (9); p<0.01. The reliability of the test showed moderate to substantial agreement (ICC = 0.74), with a small learning effect between two sessions.

**Conclusion:**

The dual-task test was sensitive enough to discriminate between elderly and young people. It revealed that the elderly did not utilize cognitive cues for their anticipatory postural control strategies as well as the young were able to. The test procedure was feasible and comprehensible for the participants, and it may be relevant to standardize a similar test for an alternative dual-task approach in the clinical setting.

## Introduction

The assessment of balance in the elderly population has high priority in order to identify individuals with postural control deficits and increased risk of falling [1]. Postural control is defined as the act of maintaining, achieving or restoring a state of balance during any posture or activity. Postural control strategies may be either predictive or reactive, and may involve either a fixed-support or a change-in-support response [2]. Balance tests should therefore assess different components of balance ability including anticipatory/feedforward balance strategies and not only postural control in a standing position [3]. Clinical tests designed for the assessment of postural control may challenge the individual’s limit of stability [4] or they may challenge the individual’s ability to change the base of support by the use of stepping strategies [5].

Postural control is often considered to be automatic and require minimal attention, but this is mainly true for a well-trained healthy individual performing a relatively easy task. There may very well be significant attention requirements for postural control, depending on the postural task, the age and the balance abilities of the individual [6]. Many tests which are used to assess physical performance and balance allow the subjects to compensate for their deficits by utilizing other control strategies (e.g., visual and/or cognitive regulation of task performance) [7]. To better detect deficits in the postural control a dual task assessment may be used. In a dual-task test, the subject will be required to perform an attention-demanding task simultaneously with a motor task (e.g., standing, reaching and stepping). Dual-task interference will occur if the available central attention resource capacity is exceeded, resulting in impaired performance in one or both tasks [8,9].

In the clinical assessment of an individual’s postural control, the standardization of the dual-task test procedure remains a challenge, however. It may be feasible to standardize and monitor the motor task, which may be monitored as the gait speed during walking, or as the sway pattern in a standing position. The secondary task, which is most often a cognitive task, is more difficult to standardize and monitor. The Stroop test has been used in settings where the subject does not have to move around [10], and the task of counting backward in sequences of three has been used as a cognitive task during walking [11]. Individuals with different arithmetic capacities may, however, be challenged differently by a counting task, the participants may prioritize the counting correctness in different ways, and the counting performance is difficult to monitor.

The purpose of this study was to evaluate a novel test of postural control, which incorporates the paradigm of the dual-task assessment approach in a new way. In this test, the postural control was evaluated as the agility or the gross motor function of the participant, with respect to performing a given task. This task was designed to challenge the participant’s limit of stability and to provoke stepping strategies. Good automatization of the dynamic postural control during the task would allow the participant to use residual attentional capacity for a secondary cognitive task. This cognitive task was to analyze and use leading cues for the anticipatory strategies. The utilization of anticipatory strategies would be revealed in a better overall performance time, when leading cues were provided. The improved performance time was a common result of both the motor and the cognitive performance. This approach was different in its nature from the traditional dual-task approach, as the two tasks were not at first measured separately and then together. It may, however, better mimic a daily life situation where the task of crossing the street, climbing a curb and paying attention to the traffic represent challenges with respect to both the postural control and the attention directed towards the task and the surroundings.

Automated postural control allows residual attentional capacity to utilize a preparatory information for anticipatory postural control strategies, and to improve performance in a given task. In this way, the proposed test evaluated whether the postural control during the primary task of standing, reaching and stepping was automated, by assessing the improvement in performance time with a leading cue.

The construct validity of the test was evaluated by its ability to discriminate between the postural control of elderly and young people. The elderly are more challenged and less automated than young people in a task which will force them to approach and exceed their limit of stability. The residual capacity for a secondary task will therefore be smaller amongst the elderly [4,5]. We hypothesized that the elderly would not be able to improve their performance time with cognitive cues for the motor task like young people would.

## Materials and Methods

Thirty-one healthy community-dwelling elderly (mean age: 77 (7) years; male: 19%) and 15 young participants (mean age: 23 (4); male: 20%) were included in this study. Fewer young than elderly people were included as a more homogenous character of group of young people was expected. The elderly participants were recruited from community centers. They were included if they were older than 65 years, independent in activities of daily living and reported no history of frequent falling within the last six months. Exclusion criteria were: dependency on walking aids, significant pain that limited daily functions, known uncorrected visual or vestibular problems, or cognitive impairment (i.e. Mini Mental State Examination (MMSE) < 23) [12]. The young adults were included if they had no known disease or need for medication. The two groups were matched on gender only.

The elderly participants answered a questionnaire to characterize their fear of falling: Fall efficacy scale (FES) [13]. The general physical performance of the elderly was evaluated by the “Timed Up and Go” test (TUG) [14]. Their mean scores were TUG: 7.5 (1.6) seconds; FES: 20 (4).

The Ethics Committee of Region North Jutland approved the protocol and the participants provided their written informed consent to participate in this study in accordance with the consent procedure of the regional ethics committee.

### Testing procedure

**Fig 1:**
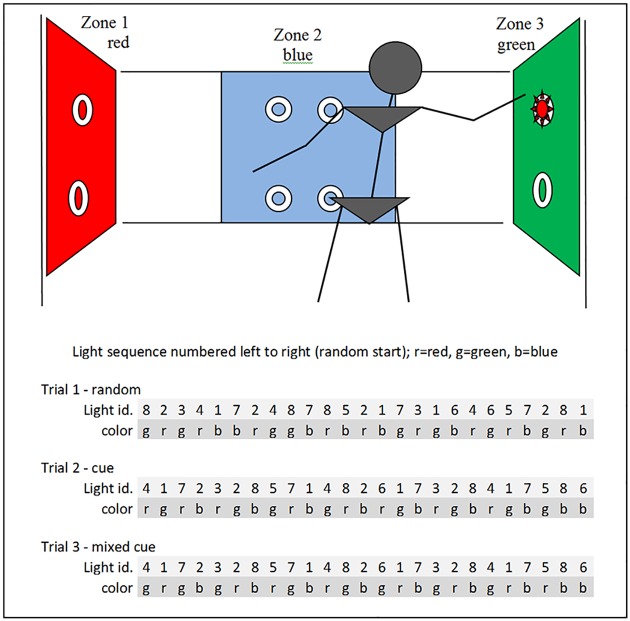
Setup. Lights/sensors were placed in three zones. Zone 1 and 3 were marked red and green respectively and they were beyond reach for a person positioned at the center of the field. The middle zone was blue. The light sequence was different in the three trials, but all lights were equally represented. In trial 2 and trial 3, the color of the light indicated the position of the following light.

Eight lights with inbuilt sensors were placed on three sides of the participant so that the participant had to challenge his/her limit of stability when reaching for the lights. Four lights were placed in a blue zone on a wall in front of the participant, two lights were placed to the left of the participant in a red zone, and two lights were placed to the right of the participant in a green zone. The pairs of lights were placed at shoulder and waist height, respectively (see Fig 1). The lights in the red and green zones were placed 0.5 meters away from the wall and three meters apart. This ensured that they were out of reach when the participant was standing in the middle of the setup.

In order to reach the lights, the participant would need to challenge his/her limit of stability while reaching out for a light; or he/she would need to change the base of support by making stepping strategies in order to move the whole body closer to the light. In this way, the participant was challenged in a variety of feedforward postural control strategies in different directions.

The dual-task test consisted of three trials with a motor task, which was the same through all the trials, and with a cognitive task, which was different between the trials in its demand on cognitive resources.

In each trial the motor task consisted of 25 repetitive reaching tasks in which the participants were to hold their hand in front of one out of eight lights/sensors in order to turn off the light. The lights were lit one at a time, and once the light of one sensor had been turned off, the subsequent sensor would light up after a 0.5-second delay. The number of activated lights in the different zones was equally divided in all trials in order to ensure that the participant was equally challenged within and beyond his/her limits of stability (Fig 1). In this way, the three different trials challenged the balance and postural control in the same way.

The three trials in the test were different in their cognitive demands. By giving different cues, the participant would be provided with different possibilities to use cognitive strategies in the tasks:

1. The lights would be lit in random order using one of three colors (red, green and blue). No cue was given as to where the next light would appear. With respect to the cognitive demand, this test would mainly be challenging the participant’s reaction time.
2. The color of the light indicated the position of the next light. This was a cue that could be utilized when the person was capable of dual tasking.
  If the light was red, the following light would be lit in the red sector. If the light was green, the following light would be lit in the green sector. If the light was blue, the following light would be in the blue sector.
3. The color of the light indicated the position of the next light, but the red and green cues were reversed. If the light was red, the following light would be in the green sector. If the light was green, the following light would be in the red sector. If the light was blue, the following light would be in the blue sector. This sequence added a cognitive load when utilizing the cues.

See the programmed sequence of lights in Fig 1. The program was started at a random point in the sequence.

The three trials were performed in the order indicated in the previous section. Before each test, the participants were introduced to the procedure and they were allowed several attempts until they felt familiar with the task. They were instructed to turn out the lights as fast as possible while still keeping a safe balance.

The whole procedure was performed twice with a break of ten minutes in between the two sessions. This was done in order to evaluate the test-retest reliability, and to allow a familiarization to the test procedure. Only data from the second session were used for the validation of the test.

The lights and the software used for the test were products from the FitLight Sports Corp. Ontario, Canada. The commercially available product, FitLight Trainer, comes as a wireless system unit comprising eight LED powered lights controlled by a tablet. The lights have an inbuilt sensor which reacts to proximity or touch and deactivates the light. The system may be programmed for specific sequences of light activation and responds to the deactivation of the lights, while the timing is recorded by the controller.

### Data analysis

The performance time for each trial was automatically recorded by the software of the FitLight system and displayed on the tablet-controller of the system. The figures were manually entered in MS Excel, and the statistical analyses were done in SPSS 22.0.

For each group and session, the averaged performance times of the trials were presented, and the relative percentage changes from trial 1 (random) to trial 2 and 3 (cue and mixed cue) were calculated. The normal distribution of data was evaluated by QQ-plot.

Reliability was evaluated by absolute differences between sessions and intraclass correlation coefficients (ICCs). ICCs were calculated with a two-way mixed effects model (ICC 3,1) using absolute agreement. These values were interpreted with the labels assigned by Landis and Koch [15], where values of 0.00–0.40 indicate unacceptable agreement, 0.41–0.60 indicate moderate agreement, 0.61–0.80 indicate substantial agreement and 0.81–1.00 indicate almost perfect agreement.

In order to avoid a possible bias due to a learning effect, only data from session 2 were used for the validation of the test. A comparison of the performance in the three trials and between groups was evaluated by factorial repeated measures ANOVA (GLM repeated measures). The scores and differences between trials were presented in graphs by their mean and confidence intervals (CI 95%). Performance improvements in trials with cues were evaluated by the changes relative to the individual baseline scores.

## Results

**Fig 2:**
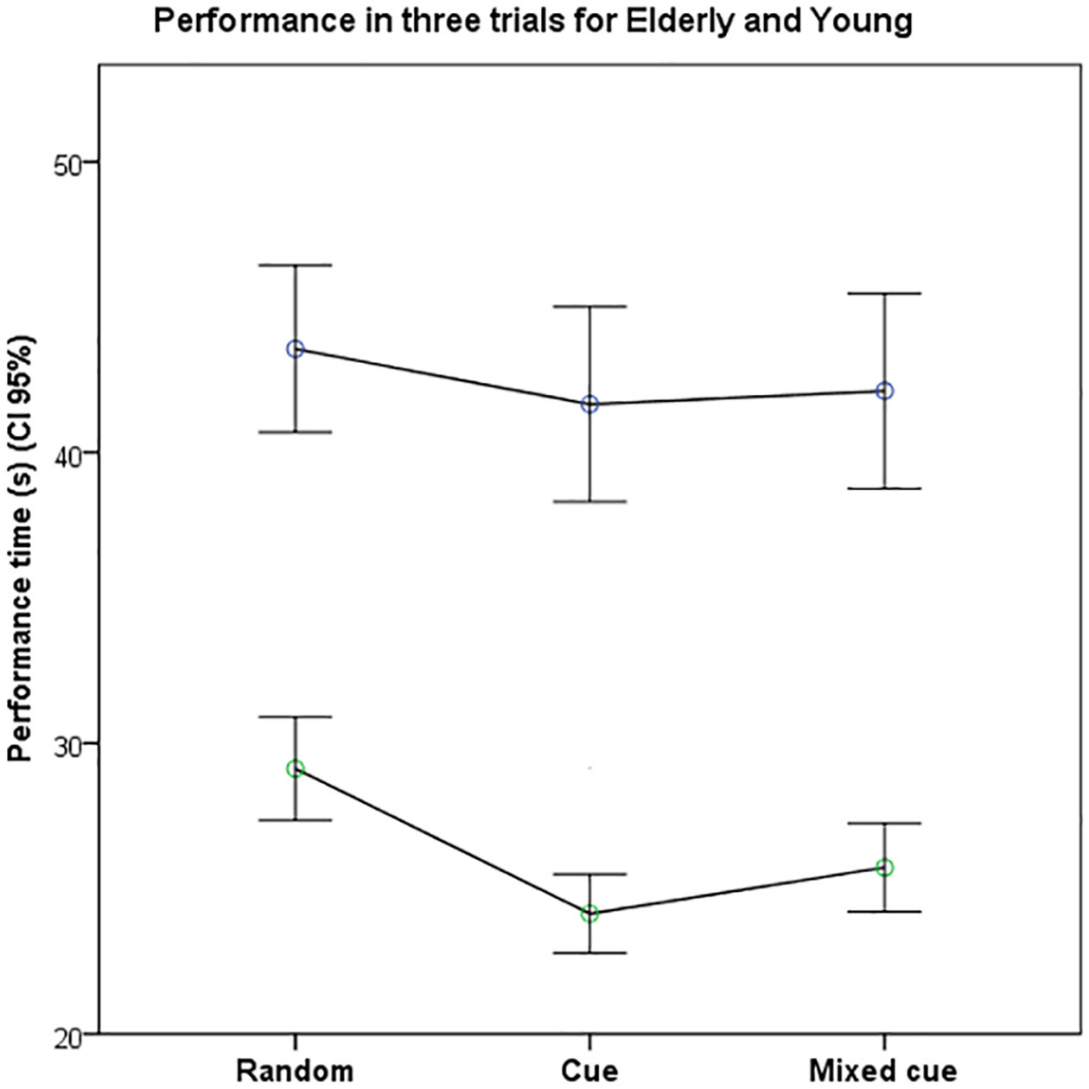
Performance time. The elderly were generally slower than the young in all trials, but both groups improved their performance (i.e. used shorter time) when they were provided with a leading cue. * p<0.05, ** p<0.001.

Both groups displayed the same pattern in their performance time over the three trials in both the first and the second session (Fig 2). Both groups improved their performance time when provided with a leading cue for the motor task. In general, the group of elderly people performed the trials significantly slower than the young group, p<0.001.

In comparison with the baseline trial, there was an improvement when a leading cue was provided. The improvement in performance time was larger in the trial with the more simple cognitive load, i.e., a cue in the right color, compared to the more complex trial with a mixed color cue.

The group of young participants improved their performance more than the elderly, and this Trial*Age group effect was statistically significant (F _(2,1)_ = 5.5; p<0.05).

Both groups performed significantly better in the cue trial, while this was only the case for the young in the mixed cue trial.

**Fig 3:**
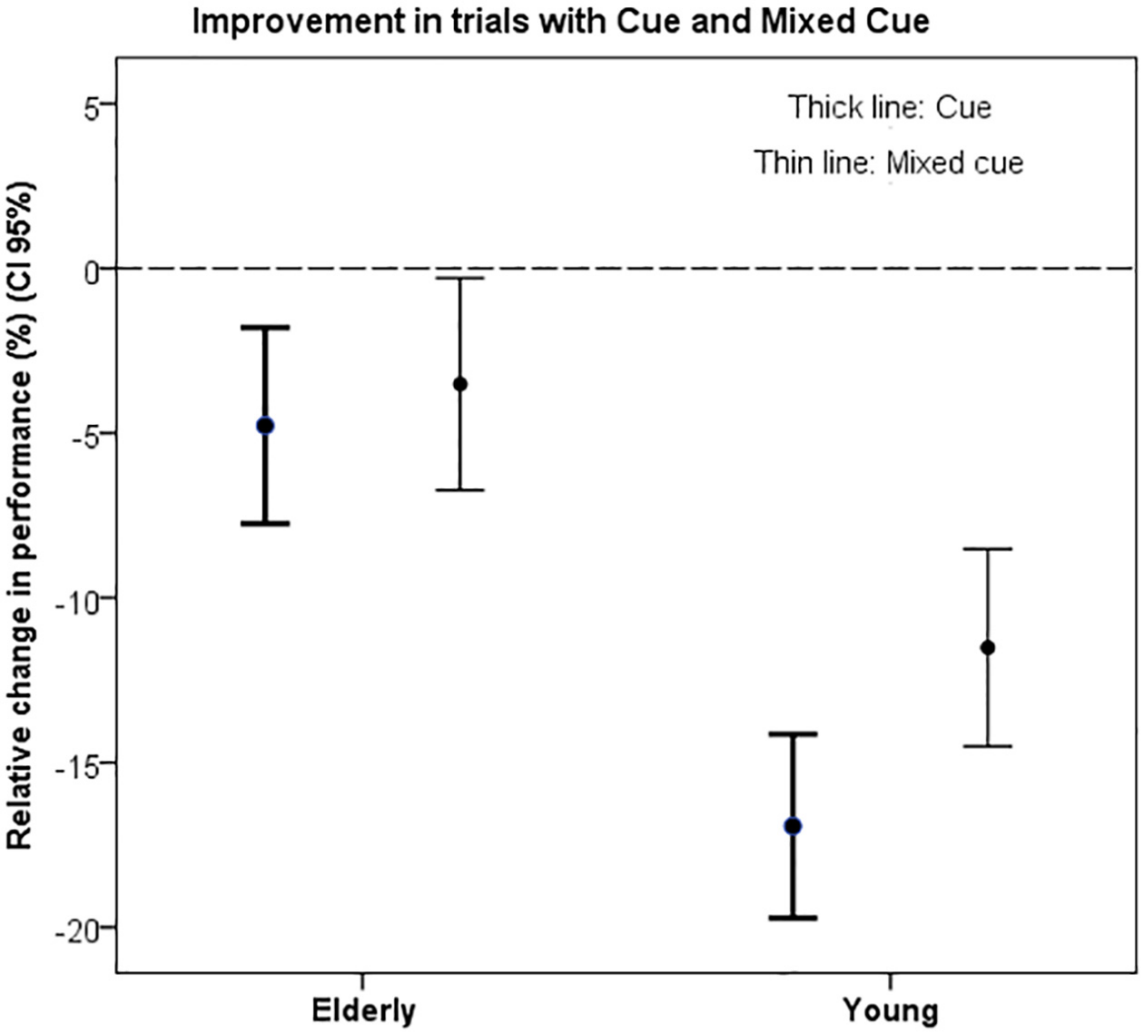
Relative improvement. The relative improvement in performance time was significantly different between groups and it was smaller in the group of elderly people. * p<0.001.

The relative improvement in performance was larger in the young group compared to the elderly group in both the cue trial (p<0.001) and the mixed cue trial (p<0.01; Fig 3).

The whole test sequence was done twice. A faster performance time was seen in all trials in both groups for the second session compared to the first session, but the difference was not statistically significant in either group (Table 1).

**Table 1:** Performance time in three trials and improvements with respect to trials with a leading cue.

	Random	Cue	Mixed Cue		Random vs Cue	Random vs. Mixed Cue
Elderly						
Session 1	46.7 (8.5)	44.9 (9.5)	44.7 (10.5)		-3.8% (9.1)	-4.3% (10.8)
Session 2	43.6 (7.8)	41.7 (9.2)	42.1 (9.2)		-4.8% (8.1)	-3.5% (8.8)
				ICC	0.51 (-0.03–0.77)	0.61 (0.19–0.82)
Young						
Session 1	29.9 (3.5)	25.6 (3.0)	26.4 (2.8)		-14.4% (4.4)	-11.3% (5.1)
Session 2	29.1 (3.2)	24.1 (2.5)	25.7 (2.8)		-16.9% (5.0)*	-11.5% (5.4)
				ICC	0.74 (0.20–0.91)	0.64 (-0.14–0.88)

Time score in seconds; mean and SD, and reduction in time score, mean percentage (SD). Negative values represent performance improvements. ICC: Intra-class correlation coefficient (CI 95%).

*p<0.05 difference between session 1 and 2.

The improvement in performance time with a leading cue was the main outcome of the test. This improvement was significantly better in the second session for the group of young people with respect to trial with cue. Intraclass correlation coefficients indicated moderate to substantial agreement.

## Discussion

In general, the motor performance in the group of the elderly was slower, compared to the group of young people. In the trials with a cognitive cue for the task performance, the young performed relatively better than the elderly did. Apparently, the elderly were not able to utilize the information in the cues as well as the young.

**Fig 4:**
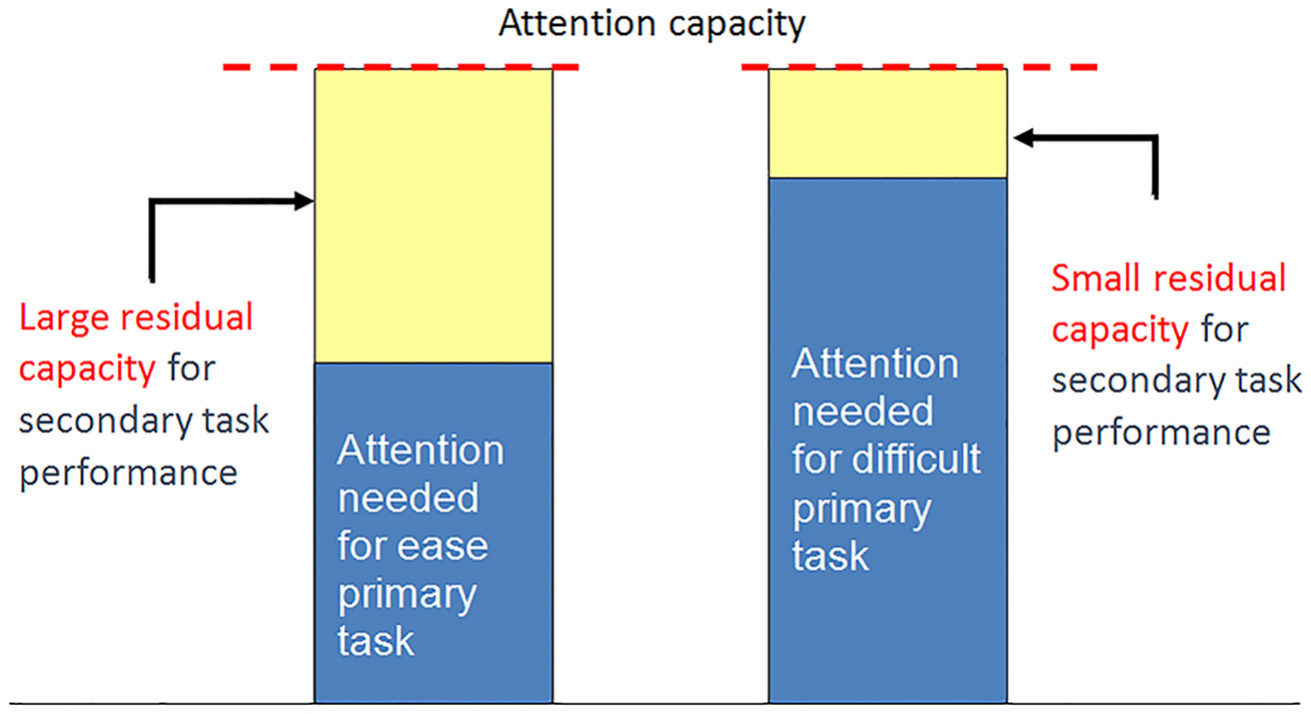
Dual task model. Illustration of the difference in residual attentional capacity for a secondary (cognitive) task in relation to an automated and a non-automated primary (motor) task. (Modified from Abernethy 1988).

It was not surprising that the elderly performed slower than the young people did. It was interesting, however, that the elderly did not reveal a comparable relative improvement in their performance time when they had the possibility to predict the position of the next light and use anticipatory postural control strategies. This may be explained by a model by Abernethy, which illustrates an attention capacity-sharing hypothesis (Fig 4). When a primary task is more demanding, a greater proportion of the individual’s processing capacity must be allocated to maintain an acceptable level of performance [16]. As the central processing capacity is limited, an attention-demanding primary motor task will result in less residual processing capacity for a secondary task [8]. A poorer performance in a secondary task may therefore indicate that a large proportion of the attention is directed toward the primary task, or that the attention capacity in general is limited.

According to the understanding of this model, the residual attentional capacity may have been insufficient amongst the elderly since they were not able to utilize the leading cues as well as the young people. The motor task may have required more attention, or the attention capacity may have been limited among the elderly. Either factor is of relevance with respect to postural control and the performance of daily activities in a distracting environment. In such a context, it is crucial that the postural control is automated to an extent where a residual attentional capacity may be available for orientation or observation of obstacles.

The organization and automatization of motor control and postural control is developed through motor learning. Fitts and Posner articulated three stages of motor learning, consisting of 1) a cognitive stage, 2) an associative stage and 3) an autonomous (automatic) stage [17]. The first stage requires conscious attention to each part of the movement, whereas the third stage leaves attention resources for other tasks. Motor tasks that are well trained and not very demanding should be autonomous and be performed with little conscious attention.

Postural control in most activities of daily living (e.g., standing and reaching) is well trained and does not place high demands on attention resources [17]. In contrast, when the motor task is difficult or when sensory or motor deficits occur, the complex generation of movement may have to be restructured. With age-related deterioration of preconditions for postural control (i.e. muscle weakness, poorer eyesight, decreased perception of high frequency vibrations, proprioception and pressure stimuli, etc.) reorganization and adjustment of the previously learned motor control may require new motor strategies. In accordance with the Fitts and Posner model, this may lead to a step-down to an associative or a cognitive stage [18,19]. When the benefits from the movement automatization are lost, the motor performance will be more attention demanding. Alternatively, the motor strategies have to be relearned to an automatic stage [20].

Mulder et al. have argued that most tests that are used to assess physical performance allow the subjects to compensate for their deficits by utilizing other control strategies (e.g. cognitive regulation of task performance) [7]. When assessing the functional capacity of a patient, focus has therefore been directed toward the interaction between cognitive factors and motor performance [16]. Dual-task paradigms may be used to investigate the attention demands of a motor task and to examine the effects of concurrent cognitive or motor tasks on motor performance [21,22]. The use of a dual task test has been suggested for fall prediction [23], but the value of this approach is also questioned [24]. In studies on Parkinson’s disease, the dual task approach has been used with some success to assess early stages of the disease [25].

The dual-task procedures for balance assessment have traditionally been designed with a specific motor task which is combined with a distracting cognitive task [11]. When the participants perform worse in the motor task with the cognitive task, this may be interpreted as a sign of poor automatization. In the present study another approach was proposed. In this dual-task protocol the participants were encouraged to use their residual attention capacity to perform better in the motor task. They were given a cue in the task, which could be utilized in order to predict where the next light would appear and allow the use of anticipatory postural control strategies. Thereby, they had the possibility to improve their performance and execute the trial faster.

As stated by Mulder et al., impaired postural control may not be revealed in a standard examination in which the residual attention capacity is not sufficiently challenged. In the test procedure of the present study, the participants sat their own benchmark for the performance, as they chose the movement velocity with respect to their own capacity. The floor and ceiling effect of the test was thereby partly eliminated. Nevertheless, it is still not possible to standardize to what extent the individual will challenge him/herself. In such a test the motivation of the participant remains an unknown factor. Therefore, the relative time-improvement with cues was used for the individual’s performance evaluation and for the comparison between groups.

It may be expected that postural control changes with age due to age-related alterations in the brain, loss of muscle mass and decreased perception of high-frequency vibrations, touch, proprioception and pressure stimuli [18,19]. Thus, it is likely that the elderly require increased conscious attention to maintain postural control in a given motor task and thereby will have less residual attention capacity for a cognitive task [10]. In this study we therefore hypothesized that the elderly were not able to use the cognitive cues in the dual-task test as well as the young, and the testing procedure was validated with respect to its ability to discriminate between young and elderly. The findings showed that performance can be improved by the use of anticipatory strategies based on cognitive cues, and that the elderly were less capable of utilizing these strategies. This is an argument for the construct validity of the test.

The findings indicated a learning effect from session 1 to session 2. The reliability of the test was moderate, as the ICCs indicated moderate to substantial agreement. It must therefore be advocated that the participants are given ample opportunity to practice the trials before the performance time is recorded.

The test was based on several trials in which the participant should turn off lights, and this seemed to be a very appealing task. Both young and elderly became very motivated to perform the task as fast as possible. A short instruction and a few trials were enough to start up the testing procedure, and no further encouragement was needed.

The lights were a commercially available standard product, and no modifications were made to the system. The setup of the lights and the programming of the light sequences were original, however.

The participants were instructed to do the test in a tempo that was safe with respect to their balance. No falls or near falls were recorded. A few of the elderly participants experienced that the trials, which could last up to one minute, were a cardiovascular challenge. One may consider limiting the number in the sequence of lights for the trial. The test would probably still be able to reveal a dual-task deficit even when there were only fifteen lights in each trial.

A limitation of the study was the lack of possibility to make a criterion validation by comparison of the test with respect to an established test, as there exists no “gold standard” for the evaluation of dynamic postural control. The test’s ability to discriminate between young and elderly people is not a strong argument for its relevance. Future studies should evaluate whether the test can identify elderly people with poor postural control who may be at increased risk of falling.

The major consideration during the design of the test was its clinical feasibility. In most dual-task tests it is difficult to standardize and monitor the cognitive task. In the proposed test, the motor task and the cognitive task are interacting, and the performance time is a common output, which is easy to monitor in a clinical setting. This is a different approach to what has been seen in other settings. McIssac et al. proposed an operating definition of dual task, distinguishing from a complex single task: Dual tasking is the concurrent performance of two tasks that can be performed independently, measured separately and have distinct goals [26]. With respect to this definition, the test in the present study is not a dual task test. This controversy is possibly evolving from differences in the aim of the dual task approach with one focusing more on basic science and the other focusing more on applied science in a clinical context and the feasibility of the dual task approach. There are arguments for the relevance of both approaches and the subject remains open for discussion.

## Conclusion

The proposed test procedure was sensitive enough to detect the expected differences in performance between young and elderly. The elderly did not utilize the cognitive cues for their anticipatory strategies as well as the young did, which indicates that the elderly needed to direct their attention capacity toward the motor task. The test procedure was comprehendible for the participants, and the protocol seemed feasible to standardize and monitor in a clinical setting.

## Supporting Information

S1 Fig Performance time. The elderly were generally slower than the young in all trials, but both groups improved their performance (i.e. used shorter time) when they were provided with a leading cue. S1 Performance time in three tasks: Random, Cue or Mixed cue.
(PDF)

S2 Fig Figure and Table. Relative improvement. The relative improvement in performance time was significantly different between groups and it was smaller in the group of elderly people. Change in performance time (percentage) when Cue or Mixed cue was provided.
(PDF)
