# Supplementary material for: The Use of Cognitive Cues for Anticipatory Strategies in a Dynamic Postural Control Task - Validation of a Novel Approach to Dual-Task Testing
Source: PLoS One. 2016 Aug 3;11(8):e0157421. doi: 10.1371/journal.pone.0157421 (PMC4972392; doi:10.1371/journal.pone.0157421)
Supplement: S1 Fig — The elderly were generally slower than the young in all trials, but both groups improved their performance (i.e. used shorter time) when they were provided with a leading cue. S1 Performance time in three tasks: Random, Cue or Mixed cue. (PDF) [file pone.0157421.s001.pdf]

	**Performance time in three tasks: Random, Cue or Mixed cue**								
	Session 1			Session 2					
id	Random	Cue	Mixed cue	Random	Cue	Mixed cue	Age	FES	TUG
1	43,8	44,0	39,9	41,6	39,3	39,7	70	17	7,3
2	48,6	49,9	46,6	49,6	49,6	49,8	89	21	8,1
3	44,5	39,2	38,7	39,5	39,1	36,9	79	16	6,9
4	57,6	57,1	48,0	43,1	41,6	41,9	86	22	9,6
5	43,7	42,4	41,4	40,8	40,7	40,0	66	33	6,1
6	57,8	53,7	51,0	53,9	52,2	53,2	75	18	8,2
7	57,8	64,9	66,9	52,3	60,2	59,9	84	18	9,1
8	45,3	55,6	47,9	47,3	50,1	47,5	77	18	8,9
9	47,4	40,6	41,2	38,2	35,3	36,0	70	16	5,9
10	37,8	42,2	43,2	37,9	34,9	38,6	68	19	7,4
11	43,2	40,3	41,3	39,9	38,8	38,7	71	20	6,5
12	45,6	42,9	43,0	40,3	44,9	48,0	75	18	7,9
13	33,3	33,1	31,4	32,2	28,8	29,8	74	17	7,0
14	36,0	34,0	32,3	33,4	30,2	34,1	66	16	5,7
15	33,7	33,0	33,1	34,0	26,7	29,8	68	18	6,4
16	47,3	41,3	39,2	40,7	37,9	36,7	80	19	9,2
17	44,4	41,2	51,2	46,1	38,0	41,5	78	17	6,3
18	41,1	41,6	38,9	36,2	36,5	37,8	84	20	5,7
19	56,9	56,5	53,1	56,7	51,4	47,4	84	29	10,0
20	73,1	72,1	82,8	66,9	69,0	74,0	82	16	9,4
21	53,1	52,9	49,1	55,6	47,8	47,3	83	29	9,2
22	53,2	47,3	50,6	47,4	50,7	50,0	94	25	8,1
23	39,0	31,7	37,0	37,9	34,5	36,0	73	18	5,8
24	35,1	34,3	31,8	33,2	29,8	29,1	67	18	4,9
25	40,6	34,8	34,3	38,7	34,0	36,1	70	16	5,3
26	47,9	45,8	46,8	47,4	42,3	42,6	75	19	8,0
27	43,2	45,0	47,2	47,0	44,5	45,1	86	22	11,7
28	48,1	47,4	56,6	42,0	42,4	42,8	75	19	7,3
29	46,4	38,8	40,3	39,8	38,9	37,0	78	16	6,9
30	50,3	46,9	42,0	43,3	40,7	41,5	78	17	6,4
31	50,8	41,8	40,4	47,5	40,5	36,7	81	21	7,5
