# Supplementary material for: The Use of Cognitive Cues for Anticipatory Strategies in a Dynamic Postural Control Task - Validation of a Novel Approach to Dual-Task Testing
Source: PLoS One. 2016 Aug 3;11(8):e0157421. doi: 10.1371/journal.pone.0157421 (PMC4972392; doi:10.1371/journal.pone.0157421)
Supplement: S2 Fig — Relative improvement. The relative improvement in performance time was significantly different between groups and it was smaller in the group of elderly people. Change in performance time (percentage) when Cue or Mixed cue was provided. (PDF) [file pone.0157421.s002.pdf]

	Change in performance time (percentage) when Cue or Mixed cue was provided											
	Session 1						Session 2					
id	Random	Cue	diff%	Mixed cue	diff_rand%	diff_cue%	Random	Cue	diff%	Mixed cue	diff_rand%	diff_cue%
1	43,8	44	0,5	39,9	-8,9	-9,3	41,6	39,3	-5,5	39,7	-4,6	1,0
2	48,6	49,9	2,7	46,6	-4,1	-6,6	49,6	49,6	0,0	49,8	0,4	0,4
3	44,5	39,2	-11,9	38,7	-13,0	-1,3	39,5	39,1	-1,0	36,9	-6,6	-5,6
4	57,6	57,1	-0,9	48,0	-16,7	-15,9	43,1	41,6	-3,5	41,9	-2,8	0,7
5	43,7	42,4	-3,0	41,4	-5,3	-2,4	40,8	40,7	-0,2	40,0	-2,0	-1,7
6	57,8	53,7	-7,1	51,0	-11,8	-5,0	53,9	52,2	-3,2	53,2	-1,3	1,9
7	57,8	64,9	12,3	66,9	15,7	3,1	52,3	60,2	15,1	59,9	14,5	-0,5
8	45,3	55,6	22,7	47,9	5,7	-13,8	47,3	50,1	5,9	47,5	0,4	-5,2
9	47,4	40,6	-14,3	41,2	-13,1	1,5	38,2	35,3	-7,6	36,0	-5,8	2,0
10	37,8	42,2	11,6	43,2	14,3	2,4	37,9	34,9	-7,9	38,6	1,8	10,6
11	43,2	40,3	-6,7	41,3	-4,4	2,5	39,9	38,8	-2,8	38,7	-3,0	-0,3
12	45,6	42,9	-5,9	43,0	-5,7	0,2	40,3	44,9	11,4	48,0	19,1	6,9
13	33,3	33,1	-0,6	31,4	-5,7	-5,1	32,2	28,8	-10,6	29,8	-7,5	3,5
14	36	34	-5,6	32,3	-10,3	-5,0	33,4	30,2	-9,6	34,1	2,1	12,9
15	33,7	33	-2,1	33,1	-1,8	0,3	34,0	26,7	-21,5	29,8	-12,4	11,6
16	47,3	41,3	-12,7	39,2	-17,1	-5,1	40,7	37,9	-6,9	36,7	-9,8	-3,2
17	44,4	41,2	-7,2	51,2	15,3	24,3	46,1	38,0	-17,6	41,5	-10,0	9,2
18	41,1	41,6	1,2	38,9	-5,4	-6,5	36,2	36,5	0,8	37,8	4,4	3,6
19	56,9	56,5	-0,7	53,1	-6,7	-6,0	56,7	51,4	-9,3	47,4	-16,4	-7,8
20	73,1	72,1	-1,4	82,8	13,3	14,8	66,9	69,0	3,1	74,0	10,6	7,2
21	53,1	52,9	-0,4	49,1	-7,5	-7,2	55,6	47,8	-14,0	47,3	-14,9	-1,0
22	53,2	47,3	-11,1	50,6	-4,9	7,0	47,4	50,7	7,0	50,0	5,5	-1,4
23	39	31,7	-18,7	37,0	-5,1	16,7	37,9	34,5	-9,0	36,0	-5,0	4,3
24	35,1	34,3	-2,3	31,8	-9,4	-7,3	33,2	29,8	-10,2	29,1	-12,3	-2,3
25	40,6	34,8	-14,3	34,3	-15,5	-1,4	38,7	34,0	-12,1	36,1	-6,7	6,2
26	47,9	45,8	-4,4	46,8	-2,3	2,2	47,4	42,3	-10,8	42,6	-10,1	0,7
27	43,2	45	4,2	47,2	9,3	4,9	47,0	44,5	-5,3	45,1	-4,0	1,3
28	48,1	47,4	-1,5	56,6	17,7	19,4	42,0	42,4	1,0	42,8	1,9	0,9
29	46,4	38,8	-16,4	40,3	-13,1	3,9	39,8	38,9	-2,3	37,0	-7,0	-4,9
30	50,3	46,9	-6,8	42,0	-16,5	-10,4	43,3	40,7	-6,0	41,5	-4,2	2,0
31	50,8	41,8	-17,7	40,4	-20,5	-3,3	47,5	40,5	-14,7	36,7	-22,7	-9,4
